# Evidence on interventions that promote the mental health and psychosocial wellbeing of migrant, refugee and asylum-seeker children and adolescents in transit: a scoping literature review

**DOI:** 10.1186/s12939-026-02812-3

**Published:** 2026-03-12

**Authors:** Zeus Aranda, Hellen Mata-González, Aurore Brossault, Yasemin Kisbu, Xinshu She, Ana Cristina Sedas, Daniel Bernal, José Pulido-Manzanero, Enrique Regidor, Anna M. Mandalakas, Karla Fredricks

**Affiliations:** 1 Universidad Complutense de Madrid, Madrid, Spain; 2 Baylor College of Medicine, Houston, TX USA; 3 Médicos Sin Fronteras, Mexico City, Mexico; 4 UNICEF México, Mexico City, Mexico; 5 Koç University, Istanbul, Turkey; 6 Stanford University School of Medicine, Stanford, CA USA; 7 Johns Hopkins University, Baltimore, MD USA; 8 Instituto Tecnológico y de Estudios Superiores de Monterrey, Mexico City, Mexico; 9 London School of Hygiene and Tropical Medicine, London, UK; 10 CIBER de Epidemiología y Salud Pública, Madrid, Spain; 11 Instituto de Investigación Sanitaria del Hospital Clínico San Carlos, Madrid, Spain

**Keywords:** Mental health, Psychosocial wellbeing, Children, Adolescents, Minors, Program evaluation, Migrants, Refugees, Asylum seekers, Forcibly displaced

## Abstract

**Introduction:**

The population of migrant, refuge, and asylum-seeking children and adolescents across the globe has increased in recent decades. These minors often undergo adverse experiences that negatively impact their mental health and psychosocial well-being (MHPWB). In light of the transient nature and multiple stressors inherent in the transit stage, this scoping review was conducted to characterize the evidence on interventions aimed at promoting the MHPWB of minors during their migration journey.

**Methods:**

Following the PRISMA-ScR guidelines, we systematically searched the MEDLINE, Embase, Global Health, APA PsycInfo, and Web of Science databases, as well as the Google Scholar search engine, for studies published between January 2010 and September 2025 that included the evaluation of some aspect of interventions aimed at promoting MHPWB in minors during transit, regardless of the language and location where the study was conducted. Systematic reviews of the literature were excluded. The data most relevant to answering the research questions were presented in tables and accompanied by a narrative synthesis.

**Results:**

Of the 1,835 unique documents identified, 28 met the inclusion criteria. Most of the interventions had been implemented in refugee camps and were aimed at school-age children and adolescents who had been forcibly displaced, mainly from the Middle East. Most of the interventions combined different approaches, such as psychoeducation and cognitive-behavioral therapy, and had a family or group approach. All interventions reported some degree of positive change on children’s MHPWB. However, few studies considered at-risk subgroups, and no interventions targeted two of the main forcibly displaced populations at the time of the study, Venezuelans and South Sudanese.

**Conclusions:**

Our study effectively describes existing interventions aimed at promoting MHPWB for minors in transit and their effectiveness and/or implementation process, as well as identifies gaps in the current evidence and lessons learned that can help improve future interventions.

**Supplementary Information:**

The online version contains supplementary material available at 10.1186/s12939-026-02812-3.

## Abstract (es)

**Introducción:**

La población de niños y adolescentes migrantes, refugiados y solicitantes de asilo ha aumentado en las últimas décadas a nivel global. A menudo, estos menores experimentan experiencias adversas que impactan negativamente sobre su salud mental y bienestar psicosocial (SMBP). Considerando la naturaleza transitoria y los múltiples estresores inherentes a la etapa de tránsito, se realizó la presente revisión de alcance para caracterizar la evidencia sobre las intervenciones orientadas a promover la SMBP de menores durante el viaje migratorio.

**Métodos:**

Siguiendo las guías de PRISMA-ScR, buscamos de forma sistemática en las bases de datos MEDLINE, Embase, Global Health, APA PsycInfo y Web of Science, así como en el motor de búsqueda Google Scholar, estudios publicados entre enero de 2010 y septiembre de 2025 que incluyeran la evaluación de algún aspecto de las intervenciones destinadas a promover la SMBP de los menores durante el tránsito, independientemente del idioma y lugar donde se hubiera realizado el estudio. Se excluyeron revisiones sistemáticas de la literatura. Los datos más relevantes para responder a las preguntas de investigación fueron presentados en tablas y se acompañaron de una síntesis narrativa.

**Resultados:**

De los 1835 documentos únicos identificados, 28 cumplieron con los requisitos de inclusión. La mayoría de las intervenciones habían sido implementadas en campos de refugiados y estaban dirigidas a menores en edad escolar y adolescentes desplazados forzados, principalmente originarios de Oriente Medio. La mayoría de las intervenciones combinaban diferentes abordajes, como la psicoeducación y la terapia cognitive-conductual, y tenían un abordaje familiar o grupal. Todas las intervenciones reportaron algún nivel de cambio positivo sobre la SMBP de los menores. Sin embargo, fueron pocos los estudios que consideraran a subgrupos en riesgo, así como no se identificaron intervenciones orientadas a dos de las principales poblaciones desplazadas forzosas en el período de estudio, la venezolana y sudsudanesa.

**Conclusiones:**

Nuestro estudio logra de forma efectiva describir las intervenciones existentes destinadas a promover la SMBP de menores en tránsito y su efectividad y/o proceso de implementación, así como identificar brechas en la evidencia actual y aprendizajes que pueden ayudar a mejorar futuras intervenciones.

## Introduction

The number of migrants, refugees, and asylum seekers has increased dramatically in recent decades globally, with nearly 304 million international migrants in 2024 (58% more than in 2005) [1], nearly 38 million refugees, and 8 million asylum-seekers [2]. Among the factors that contribute to the displacement of these populations are armed conflicts, organized crime, political persecution, difficulties related to climate change such as extreme weather events or decline in agricultural, livestock and fisheries productivity, economic scarcity, or a lack of infrastructure and basic services [3]. In response to these situations, many people flee to other regions to try to find safety, meet basic needs (including food and housing), obtain services such as health care and education, and improve their incomes [3].

Among migrants, refugees, and asylum seekers, there are increasing numbers of minors who travel with their families or, in many cases, unaccompanied. In 2020, it was estimated that 13% of the world’s migrant population was minors [4]. 2023 saw the highest numbers in history of international migrant children (35.5 million), refugee or asylum-seeking children (17.5 million), and children displaced in their own country due to conflict, violence and disasters (29.7 million) [5]. This population, in addition to its irregular migratory status at times and the conditions of displacement itself, is in a particularly vulnerable situation due to its young age, as they are still in the process of physical, emotional, and intellectual development [6, 7]. Among the risks faced by this population are human trafficking, related to forced labor or of a sexual nature; assaults of different kinds, including sexual assaults; exposure to other types of violence; the impact of climate agents; or exposure to pathogens or dangerous animals in transit areas [8, 9].

The difficulties faced by this population are present throughout the entire migration process, including the place of origin, the displacement phase, and the destination [10, 11]. These experiences contribute to the psychological and emotional distress of children and adolescents, with impacts that can last into adulthood, undermining their lifetime health and wellness, employment and contribution to society [12–14]. Therefore, it is necessary to implement effective programs that ensure the mental health and psychosocial well-being of minors throughout the different stages of migration, which help prevent the emergence of mental health concerns and treat them when they are already present.

Despite the implementation of various programs to promote the mental health and psychosocial well-being along the different migratory routes, implementation evidence on these interventions is still very scarce [15, 16]. This leads many organizations and researchers to design and implement independent programs from scratch, leading to inefficient use of resources and compromising the scope of these interventions [15]. It can also drive the use of inappropriate approaches—such as overly clinical or pathologizing models that are not aligned with mental health and psychosocial support best practices—potentially causing harm, stigmatization, or unintended negative effects for children, adolescents, and their families [17]. This lack of evidence is especially evident in the period of transit, which often takes place in spaces with high participant attrition and little research capacity due to lack of resources [18]. Most of the evidence is at destination, especially in high-income countries such as the United States, Canada, and some Western European countries [15]. However, the context of transit presents a series of particularities that differ from those in countries of destination and that make it uniquely important to characterize interventions aimed at this context. Among these are a prevalent sense of uncertainty, the temporary nature of stays, safety concerns, and greater difficulties in accessing basic services such as education, health or child protection services [18].

Considering the need for implementers to have access to a compendium of all the current evidence on interventions that promote the mental health and psychosocial well-being of children and adolescents in transit, as well as the need for researchers to identify gaps in the evidence where there is a greater need for evaluation, we decided to carry out this scoping review.

## Methods

The scoping review was carried out in accordance with the methodological framework defined by Arksey and O’Malley [19], subsequently extended by Levac et al. [20], and with the manual of the reviewer of the Joanna Briggs Institute (JBI) [21]. The study adheres to the PRISMA-ScR guidelines (Preferred Reporting Items for Systematic Reviews and Meta-Analyses extension for Scoping Reviews) [22]. Details of compliance can be found at Additional file 1. The protocol was published in Open Science Framework platform [23]. Artificial intelligence was not used in this scoping review.

### Data sources and searches

Primary and secondary research questions were developed using the “Population, Concept, and Context” framework suggested by the JBI Scoping Review Manual [24]. In our study, the population was “migrant, refugee, and asylum-seeking children and adolescents”; the concept was “evidence on interventions that promote mental health and psychosocial well-being”; and the context was “the transit situation at a global level”.

The primary research question was “What is the evidence on interventions that promote the mental health and psychosocial well-being of migrant, refugee, and asylum-seeking children and adolescents in transit globally?” Secondary research questions allowed us to focus on specific aspects of the interventions and the beneficiary populations and were “Where were these interventions carried out?”, “What were the sociodemographic characteristics of the beneficiaries of these interventions?“, “What were the target outcomes and approaches used in these interventions?“, “Who facilitated them?“, “What designs and methodologies have been employed to generate evidence on the interventions?“, “What were the sample sizes of these studies?“, “What were the results of these studies?“, “What were the barriers to implementing these interventions?”, and “What were the implementation enabling factors?”

We searched for articles published in peer-reviewed journals as well as reports of gray literature. For articles published in peer-reviewed journals, the Ovid MEDLINE, Ovid Embase, Ovid Global Health, Ovid American Psychological Association (APA) PsycInfo, and Web of Science databases were searched [25]. Table 1 shows an example of the search strategy that was used with the Ovid databases. Keywords based on the primary research question were searched in the title, abstract, and subject headings of the publications.

**Table 1:** Search strategy for the Ovid databases (MEDLINE, Embase, Global Health and APA PsycInfo)

Search	Query
1	(immigrant* or migrant* or asylum seeker* or refugee* or displaced).ab, kf, ti.
2	(intervention* or program* or service* or treatment* or therap* or prevent* or promot*).ab, kf, ti.
3	(psych* or emotional or mental or distress* or stress* or anxi* or depress* or post? traumatic or ptsd or trauma*).ab, kf, ti.
4	(child* or adolescent* or teenager* or minor* or youth*).ab, kf, ti.
5	(transit or on the move or mobility or temporary or shelter* or camp* or migrating or migration).ab, kf, ti.

In addition, bibliographic references of the included literature were reviewed to identify additional studies. For gray literature, a search was carried out in Google Scholar and the first 10 pages of results were screened in order according to relevance. Only the first 200 documents were screened, considering the decreasing relevance of the results and the importance of having a manageable number of documents to be screened by the study authors. This is a reference number used in other scoping literature reviews [26, 27]. The final search of all sources was conducted on September 15, 2025. All searches were conducted in English. Items were transferred to the Rayyan platform [28], where duplicate cases were removed with the automatic “de-duplicate” option.

### Inclusion and exclusion criteria

We included documents that (1) had been published since 2010 (to capture evidence as relevant as possible for the current moment but that included responses to some of the major refugee mobilizations of the 21st century, such as the one resulting from the Syrian civil war [2011], the conflict in the Central African Republic [2012], the civil war in South Sudan [2013], the Libyan civil war [2014], the crisis in Venezuela [2014], the civil war in Yemen [2014], the Rohingya genocide in Myanmar [2017], the war between Ukraine and Russia [2022], or the war between Israel and Gaza [2023], among others) [29–31], (2) had been published in any language (documents in languages other than English and Spanish were translated with the help of translation software), (3) included the evaluation of some aspect of interventions aimed at promoting the mental health and psychosocial well-being of the migrant, refugee, or asylum seeker population under 18 years of age in transit (understanding evaluation as “the systematic collection of information about the activities, characteristics, and outcomes of interventions to make judgements about the intervention, improve intervention effectiveness, and/or inform decisions about future intervention development” [32]), (4) whose implementation had been carried out anywhere in the world. Any document that did not meet these criteria was excluded from review. In addition, systematic reviews of the literature were excluded, although potentially eligible studies were searched in the list of references. Studies could include young populations over the age of 18, provided that those under the age of 18 were listed as the primary beneficiaries of the interventions evaluated.

For the purposes of this study, the migrant, refugee, or asylum-seeking population in transit were considered when they were in a location on a temporary basis (on the street, in shelters, in migrant detention centers, or in refugee camps) without the intention of settling. Despite the difficulty of providing a specific definition, the definition used in this study is in line with that established by the Office of the United Nations High Commissioner for Human Rights [18]. Regarding the operationalization of the term, all places that explicitly expressed it in the study or those where temporary stay is an intrinsic part of their nature (e.g., transit camps, refugee reception centers, and temporary shelters) were considered transit places. However, the application of the criterion was not a priori so obvious for places such as refugee camps with populations sheltered for long periods of time or settlements. Ultimately, studies involving populations living in refugee camps were included, regardless of the length of their stay, while those involving populations living in settlements were excluded. As discussed in the Forced Migration Online Thematic Guide “Camp versus settlements” [33], some of the main differences between refugee camps and settlements, which give the former a sense of temporariness, are that: in the former, the refugee population is generally segregated from the rest of the population, they are overcrowded, the housing is very precarious, the people who live there have to share some facilities, they experience a greater lack of privacy and control, their freedom of movement is restricted, and they depend on the aid provided by the institutions that manage the camp to survive, as have fewer opportunities to participate in economic activities.

### Study selection and data extraction

Two reviewers independently assessed the titles and abstracts identified in the initial search according to the inclusion criteria (ZA and HMG). In case of discrepancies between the reviewers, they reached a consensus through discussion and the participation of a third reviewer if necessary. The documents included in this first stage were analyzed in their entirety for their definitive inclusion among the selected studies, following the consensual process described above.

Two reviewers used a standardized data extraction form (ZA and HMG). This form included the authors of the document, the year of publication, the type of document, the implementing country, the target population of the intervention, a brief description of the intervention (including target outcomes and approaches used), the implementing agent, the type of the evaluation, the methodological approach of the evaluation, the number of beneficiaries of the intervention/participants in the evaluation, the evaluation results, implementation barriers, and implementation enabling factors. Each reviewer performed a quality check on the other reviewer’s extractions. In case of disagreements, they were resolved through the discussion of the two reviewers, or the opinion of a third reviewer was requested.

The data extracted from the selected documents was presented in tables and accompanied by a narrative synthesis to relate the findings to the research questions.

## Results

### Search results

**Fig. 1:**
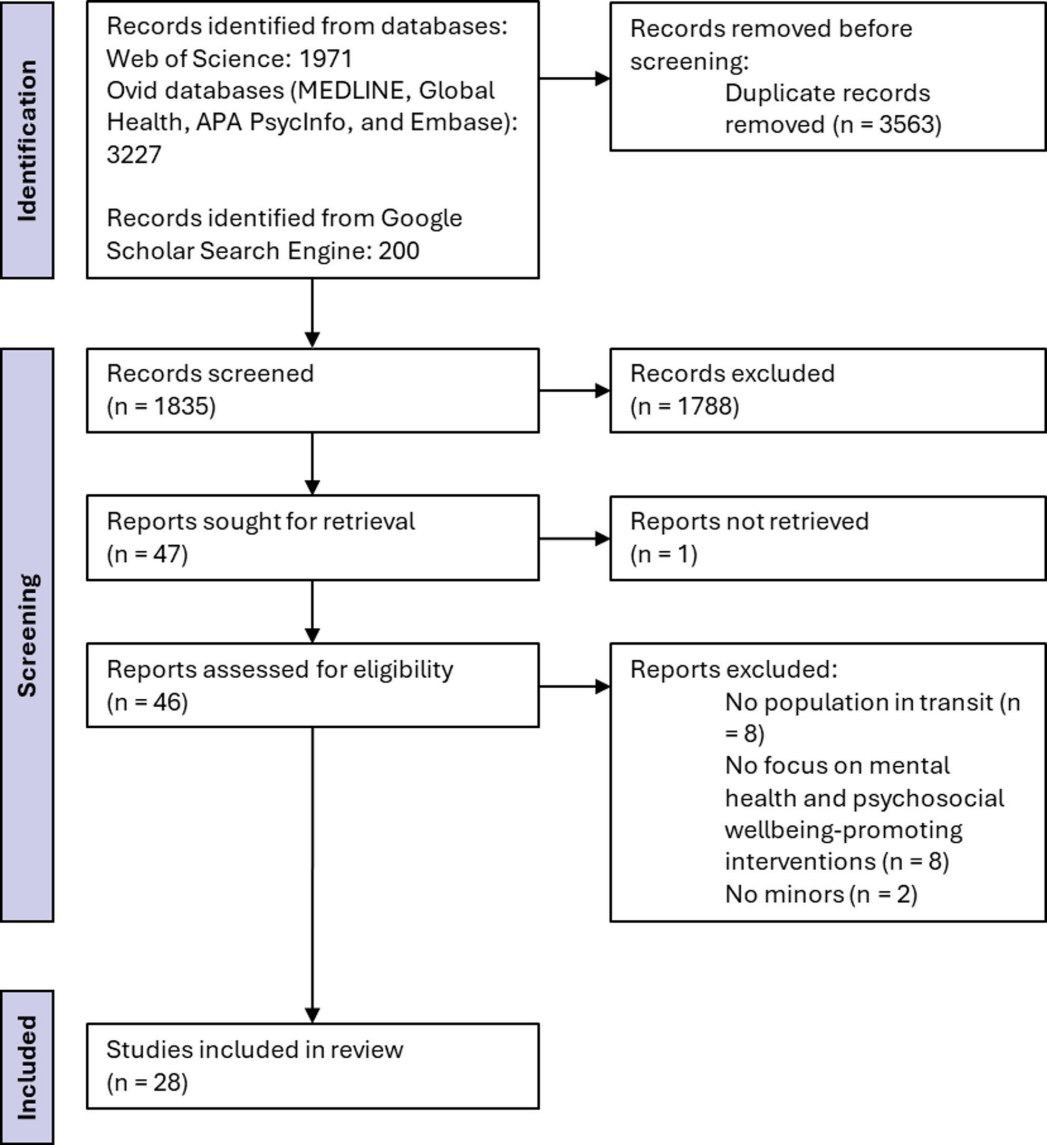
Preferred reporting items for systematic reviews and meta-analyses (PRISMA) flowchart

We identified a total of 5,398 documents, which were reduced to 1,835 after the removal of duplicates. A further 1,788 studies were excluded after reviewing titles and abstracts. One of the documents identified by the databases could not be found for review of the full document. After reviewing 46 full papers, a further 18 papers were excluded, resulting in the final selection of 28 papers for the scoping review. More details of the process can be found in the PRISMA flowchart in Fig. 1. Two of the studies report on the same intervention [34, 35], although with different approaches, so both were included as independent studies for narrative synthesis.

### Type of document and year of publication

The vast majority of the 28 selected documents (93%) had been published as original research in peer-reviewed journals, with only two exceptions, a book chapter and a conference proceeding describing the intervention, its feasibility, and its impact [36, 37]. A summary of the characteristics of the studies included can be seen in Table 2. 

**Table 2:** Summary of the characteristics of the documents included and the interventions described in them

Author and reference	Title	Year of publication	Document type	Implementing country	Characteristics of the participants	Description of the intervention (target outcome and approach)	Implementing agent
Adamkis [52]	Promoting physical activity for mental health in a refugee camp: the Skaramagas project	2022	Peer-reviewed article	Greece	Refugee minors from Syria, Iraq, Afghanistan, and Iran (12-17 years old) in refugee camp. 2.5 years of school lost on average and some without formal education.	Intervention to promote mental health and psychosocial well-being through physical activity and participation in sports. Fostering autonomy, empathy, respect for the rights of others, cooperation, and leadership skills. Combination of football, basketball, volleyball, and resistance and weight training. Three times a week for 50 minutes.	Specialized physical educator with the assistance of a school psychologist.
Akoyunoglou et al. [36]	Community music therapy with refugee children in transit camps on the Greek island of Chios: 'Like one family, together'	2020	Book chapter	Greece	Minors from Syria, Afghanistan, Palestine, Iraq, and other countries (2-14 years old) living in transit camps.	Music therapy intervention. One/two sessions per week. Focus on improving feelings of safety, fostering togetherness, connecting children through participation in musical activity in community, and strengthening their voices through song. Carrying out activities such as drawing, dancing, singing, and games. At a later stage of the intervention, community-oriented musical activities guided by trauma-informed approaches.	Four educators, a music teacher, and a music therapist.
Alem et al. [47]	Programming to Address Suicidal Behaviour among Unaccompanied Refugee Minors in a Camp Setting: A Field Report from Ethiopia	2021	Peer-reviewed article	Ethiopia	Unaccompanied and separated children in refugee camps for Eritreans, their caregivers, and other community actors.	Public awareness of suicidal ideation and behavior in minors and stigma reduction campaigns, psychoeducation for unaccompanied and separated children and their caregivers, capacity building among community members, individual and group counselling for children, and crisis response and postvention.	Paraprofessional psychosocial counsellors trained in trauma-informed developmental psychology (Eritreans) and supervision by professional psychotherapists (Ethiopians).
Bleile et al. [63]	Process evaluation of TeamUp: a movement-based psychosocial intervention for refugee children in the Netherlands	2021	Peer-reviewed article	Netherlands	Minors (6-17 years old) living in different refugee reception centers.	Movement-based psychosocial intervention (group games, sports activities, creative movement, and body awareness practices) with the aim of offering children safety, normality, and structure. Facilitators provide the space for children to release stress and tension in their bodies while offering opportunities for social interaction and resource building, with the ultimate goal of promoting their well-being and resilience.	Volunteers selected from the vicinity of the centers, of legal age, with relevant training (education, psychology, etc.), previous activity facilitating activities for children, with strong communication, interpersonal and intercultural skills, and who committed themselves for a minimum of nine months to the program. They had two-day training in a workshop, based on experiential learning.
Borja Jr. et al. [40]	Child-centred, cross-sectoral mental health and psychosocial support interventions in the Rohingya response: a field report by Save the Children	2019	Peer-reviewed article	Bangladesh	Rohingya children and adolescents in a refugee camp.	Emergency initial response of community psychosocial support (psychological first aid, structured play, sports and games for children, and psychoeducation for caregivers and teachers). Evolution to intersectoral response of child protection, education, health, and nutrition. Protection: family tracing and reunification and psychosocial support in child-friendly spaces. Education: protective learning environment and emotional and social education. Nutrition: Educating young mothers about appropriate care practices for their babies. Health: evaluation of mental health problems, psychoeducation, and counseling. Mental health promotion and education, mental health case identification and referral, and follow-up by community health workers.	Psychosocial support officers, teams specialized in the management of at-risk mothers and children and the feeding of young children in emergencies, and community health workers.
Callaghan et al. [58]	Fostering Prosociality in Refugee Children: An Intervention With Rohingya Children	2024	Peer-reviewed article	Bangladesh	Rohingya minors (5-12 years old) living in a refugee camp, born in Myanmar or in the camp.	Sessions focused on prosocial behaviors and cognitive-affective processes, based on collaborative activities, emotional perspective-taking, and executive function skills training. Small group sessions for 10 days (three hours a day) with the same partner throughout the intervention.	Rohingya researchers.
Clement-Lawrence et al. [59]	Trauma-focused counselling and social effectiveness skills training interventions on impaired psychological functioning of internally displaced adolescents in Nigeria	2020	Peer-reviewed article	Nigeria	Minors (11-14 years old) in IDP camp.	Trauma-focused counseling and social effectiveness skills training. Eight group sessions.	Two research assistants and professors at the displacement camp school who interpreted and moderated the sessions.
de Freitas-Girardi et al. [62]	Creative expression workshops as Psychological First Aid (PFA) for asylum-seeking children: An exploratory study in temporary shelters in Montreal	2020	Peer-reviewed article	Canada	Asylum-seeking children and adolescents (2-18 years old) in temporary shelters.	Creative expression workshops for asylum-seeking children and adolescents implemented as an intervention of Psychological First Aid. Socio-emotional support for children and their parents. Flexible but ritualized session structure, communication of reassuring messages to children, activities to familiarize children with the environment, creation of time and space to facilitate interactions between children, parents and facilitators, resilience-oriented workshops, and supportive and play environment, giving children the possibility to validate their experiences. Activities twice a week with a duration of one/two hours.	Eight facilitators with different career backgrounds (psychology, art, and education). They were trained and met monthly under the supervision of a person experienced in interventions of creative expression with young refugees.
El-Khani et al. [48]	Assessing the Feasibility of Providing a Family Skills Intervention, “Strong Families”, for Refugee Families Residing in Reception Centers in Serbia	2021	Peer-reviewed article	Serbia	Minors (8-15 years old) and their caregivers, from Afghanistan, refugees, living in reception centers.	Prevention intervention designed to improve parenting skills, children's well-being, and family mental health. Three sessions, five hours in total, over three weeks. Caregivers explore the strengths and abilities they have, develop better ways to cope with stress, learn tools to show children that they care about them, and learn how to encourage good behavior in their children. Children learn to cope with stress, do activities about rules and responsibilities, and think about future goals, as well as the important role of caregivers in their lives. In the joint sessions, caregivers and children learn about family values and practice sharing appreciation of others, positive communication, and de-stressing techniques.	People with direct contact with the beneficiary population in the reception centers. Non-professionals, without particular expertise. They received three-day training.
El-Khani et al. [49]	Implementation of a Family Skills Programme in Internally Displaced People Camps in Kachin State, Myanmar	2025	Peer-reviewed article	Myanmar	Families living in IDP camps.	Brief family skills intervention. Group, sessions for children and caregivers independently and as a family. Caregivers attend sessions for three consecutive weeks, and the children join in weeks two and three. One-hour sessions.	Six field facilitators, with experience working on IDPs and program-specific training.
Ertl et al. [43]	Community-Implemented Trauma Therapy for Former Child Soldiers in Northern Uganda A Randomized Controlled Trial	2011	Peer-reviewed article	Uganda	Former child soldiers with PTSD from a population survey of 1113 North Ugandans (12-25 years old) in IDP camps.	Narrative exposition intervention. Eight sessions of 90–120 minutes, three times a week. It begins with psychoeducation on PTSD and continues with narrative exposure therapy, where the participant constructs a chronology of their life to reconstruct fragmented memories of traumatic events and to achieve habituation.	Trained non-professional therapists.
Fine et al. [34]	Improving mental health in low-resource settings: A feasibility randomized controlled trial of a transdiagnostic psychological intervention among Burundian refugee adolescents and their caregivers	2021	Peer-reviewed article	Tanzania	Burundian minors (10-14 years old) and their caregivers living in refugee camp.	Intervention to reduce symptoms of internalizing disorders, including depression and anxiety, in adolescents in contexts of adversity. Seven weekly 90-minute group sessions. They focus on providing evidence-based cognitive behavioral strategies including psychoeducation, stress management, behavior activation, problem-solving, and relapse prevention. The intervention also includes three two-hour sessions with caregivers, focusing on psychoeducation, active listening, slow breathing, positive parenting strategies, self-care, and relapse prevention.	Adult refugees with at least a high school education and experience implementing programs with Burundian youth in the education sector. They received eight-day training.
Foka et al. [53]	Promoting well-being in refugee children: An exploratory controlled trial of a positive psychology intervention delivered in Greek refugee camps	2021	Peer-reviewed article	Greece	Forcibly displaced minors from Syria, Iraq, Afghanistan, Lebanon, and Kurdistan (7-14 years old) in three refugee camps.	Brief preventive group intervention aimed at war-affected children for resilience building and improved well-being. The content of each session is built around a concept of positive psychology. Psychological resources—such as optimistic thinking and promoting a sense of community and belonging—are cultivated to facilitate the experience and recollection of positive emotions and facilitate future planning, identify the character strengths of the child and the group, and improve self-esteem and mindfulness. Six two-hour sessions over a six-day period.	Three facilitators with three-day training.
Gotseva-Balgaranova et al. [50]	Impact evaluation of the evidence-based trauma stabilisation programme for refugee, asylum seeking and immigrant families	2020	Peer-reviewed article	Bulgaria and Germany	Families from Iraq, Afghanistan, and Syria (with children aged 6-11 years) residing in camps and refugee centers.	Program oriented to parent-child pairs. Objective to support parents by improving attachment with their children, while providing stabilizing techniques and psychoeducation in trauma management. It employs children's language, play, and friendly stabilization techniques. Duration between three and eight weeks. It includes nine sessions: four on parent-only psychoeducation (general trauma management issues, using psychodrama and action method techniques for concretization and visualization) and five on stabilization with parent-child pairs (based on the psychodrama-with-children approach).	Psychotherapists, social workers, educators, and psychologists.
Gul et al. [60]	Management Of Self-Concept, Disruptive Behavior And Aggression Through Art And Behavior Therapy Among Internally Displaced Children	2021	Peer-reviewed article	Pakistan	IDP minors (10-15 years old) in refugee camp.	Art therapy and progressive muscle relaxation technique of behavior therapy for two months. For art therapy, the Trauma Intervention Program for children and adolescents was used. It is a brief intervention of 8-10 sessions. Each session has its own goals that the therapist achieves through drawing activities and interviews.	Professional therapist.
Kalantzi-Azizi et al. [37]	Presentation of an online programme for children refugees: Pilot study in Greece	2017	Conference proceeding	Greece	Afghan minors forcibly displaced by the war (10-15 years old) in a refugee camp.	Online adaptation of the illustrated book "The Child and the Liberation from the Shadow of the Terrible Great Fear" of self-help for trauma for parents and children displaced by war.	NA
Kuru et al. [54]	Social-emotional outcomes in refugee children: A pilot randomized controlled trial of a school-based mindfulness intervention implemented in a refugee camp	2024	Peer-reviewed article	Türkiye	Refugee preschool children, mainly Syrians of Turkmen origin, affected by the war, living in a refugee camp.	Intervention of mindfulness practices modified according to age. The aim is to help refugee children change relationships that cause them psychological distress. Group activities consist of an introductory session followed by 12 sessions of 45–50 mins over six weeks. Each session includes mindfulness exercises, play activities, relaxation, and evaluation.	Facilitation by a person with theoretical and academic knowledge on the literature on development in at-risk children. Monitoring by a certified supervisor as a mindfulness trainer. Sessions examined by three academic experts in early childhood education.
Lakkis et al. [46]	A Pilot Intervention to Promote Positive Parenting in Refugees from Syria in Lebanon and Jordan	2020	Peer-reviewed article	Lebanon and Jordan	Syrian parents of children aged 0-6 in three refugee camps.	Intervention to positively influence parenting behaviors and achieve favorable outcomes in children. It includes topics such as nutrition, hygiene, and health; communication; positive behavior reinforcement; as well as psychosocial support sessions. It uses brainstorming activities, group work, roleplay, case studies, short presentations, and focus group discussions. Twenty-one weekly sessions of 2/3 hours.	One man and one woman per center, psychologists with experience in parenting training.
Melogno et al. [56]	War refugee children: an intervention based on coping strategies	2023	Peer-reviewed article	Italy	Refugee minors from Ukraine (7-12 years old) housed on a university campus.	Psychoeducational intervention to promote a resilient attitude in children displaced by war. Intervention of three sessions focused on the joint reading of a story for children, entitled "The perfect moment". Children engage in the story at different levels of representation, including decoding the images in the book, body language, and naming actions explicitly using the verbs "do," "say," and "think" in their native language.	Two Italian psychologists. Four bilingual Ukrainian-Italian mediators.
Metzler et al. [41]	Educational, psychosocial, and protection outcomes of child- and youth-focused programming with Somali refugees in Dollo Ado, Ethiopia	2019	Peer-reviewed article	Ethiopia	Minors (6-17 years old) in a refugee camp.	Implementation of learning centers for children and young people. In line with the characteristic approach of safe spaces for children and adolescents in humanitarian contexts. Functional literacy and numeracy skills are developed in Somali while including psychosocial activities, such as cultural dance, drawing, recreational play, and singing. Counseling is also provided. Each center offers a morning session and an afternoon session, of two/three hours for children of different ages.	Animators recruited from the field and the local community (some with teaching experience), trained, and regularly supervised.
Meyer-Demott et al. [38]	A controlled early group intervention study for unaccompanied minors: Can Expressive Arts alleviate symptoms of trauma and enhance life satisfaction?	2017	Peer-reviewed article	Norway	Unaccompanied asylum-seeking minors from Afghanistan, Somalia, Iran, Western Sahara, Palestine, and Algeria (15-18 years old) newly arrived at the reception center for asylum seekers.	Manualized group intervention of expressive arts. Ten sessions of an hour and a half spread over five weeks. Manual based on safety, stabilization, anxiety and stress management, building skills for emotion regulation, and trauma education. Sessions include a welcome, positioning on an emotion barometer, and 10-15 minutes of breathing exercises. Two cycles of five sessions focused on connecting with the group, calming down and identity, as well as hope, self-efficacy and connection.	Expressive arts therapists under the supervision of the project leader, with the support of a member of the reception center and interpreters.
Murray et al. [39]	An evaluation of a common elements treatment approach for youth in Somali refugee camps	2018	Peer-reviewed article	Ethiopia	Somali children (7-18 years old) in three refugee camps on the Ethiopian-Somali border and their caregivers.	CETA developed for the comorbid presentation of depression, anxiety, traumatic stress, and externalization of symptoms in children. 13 sessions or less.	Non-professional facilitators.
Nakkash et al. [44]	Process evaluation of a community-based mental health promotion intervention for refugee children	2011	Peer-reviewed article	Lebanon	Minors (11-14 years old) living in a Palestinian refugee camp in Beirut.	A community-based intervention for building social skills for children, parents, and teachers aimed at promoting the mental health of refugee children and their attachment to school. It began as an intensive two-week intervention in the summer and continued with a weekly session on the weekend. To achieve the objective, intermediate results such as communication skills, problem-solving skills, and the relationship with parents and teachers are changed. Intervention framed by ecological approach and community participatory research. 45 sessions with the children, 15 sessions with the parents, and 6 workshops with the teachers. Intervention informed by stress inoculation training, improving social awareness and social problem solving, and positive youth development.	Master trainer, six facilitators, and 23 young mentors. The mentors were residents of the refugee camp where the study was implemented or adjacent.
Perilli et al. [42]	EMDR group treatment of children refugees-A field study	2019	Peer-reviewed article	Türkiye	Syrian refugee minors (3-18 years old) in an orphanage that housed refugee children and adults.	Intervention based on EMDR. Participants focus on distressing memory, with its cognitive, affective, sensory, and perceptual components, while performing eye movements or exposing themselves to bilateral stimulation, using the "Butterfly Hug" in this case. Three sessions.	There were three therapists from the Italian EMDR Association, two Syrian cultural mediators residing in the orphanage who served as translators, and a Syrian psychiatrist who supervised the program.
Scheiber et al. [51]	Resilience training for unaccompanied refugee minors: A randomized controlled pilot study	2025	Peer-reviewed article	Germany	Unaccompanied male minors from Afghanistan and Pakistan (14-17 years old) residing in a shelter during the asylum procedure.	Resilience training program of six 90-minute sessions over six weeks. It includes psychoeducation, development of personal and cultural resources, and emotional regulation strategies.	Translators, clinical psychologists, and psychosocial professionals with training in trauma therapy.
Singh et al. [35]	Cultural adaptation of a scalable psychological intervention for Burundian refugee adolescents in Tanzania: a qualitative study	2021	Peer-reviewed article	Tanzania	Burundian adolescents in refugee camps and their parents.	Described in Fine et al.	Trained and supervised lay providers.
Thierrée et al. [55]	Trauma reactivation under propranolol among traumatized Syrian refugee children: preliminary evidence regarding efficacy	2020	Peer-reviewed article	Syria	Minors (7-14 years old) seeking treatment in refugee camps.	Participants were given propranolol 90 minutes before briefly recalling (reactivating) a personal traumatic memory for five consecutive days.	Professional therapist.
Weisz et al. [57]	Effects of a brief digital problem-solving intervention on depression and anxiety symptoms in Ukrainian children and adolescents displaced by war: a crossover, randomised controlled trial	2025	Peer-reviewed article	Poland	Children and adolescents displaced by Russia's invasion of Ukraine (10-18 years old) attending Polish schools.	Digital mental health intervention (on personal devices such as phones or tablets) to reduce internalizing symptoms. A single, self-guided, 30-minute session that teaches a systematic problem-solving strategy.	At each school, a study coordinator worked with teachers to implement the procedures in the classrooms.

CETA: Common Elements Treatment Approach; EMDR: Eye Movement Desensitization and Reprocessing; IDP: Internally Displaced Person; PFA: Psychological First Aid; PTSD: Posttraumatic Stress Disorder

Most of the documents were published in the period 2020–2025 (71%), followed by 2015–2019 (21%) [37–42], and 2010–2014 (7%) [43, 44].

### Place of implementation of the intervention

**Fig. 2:**
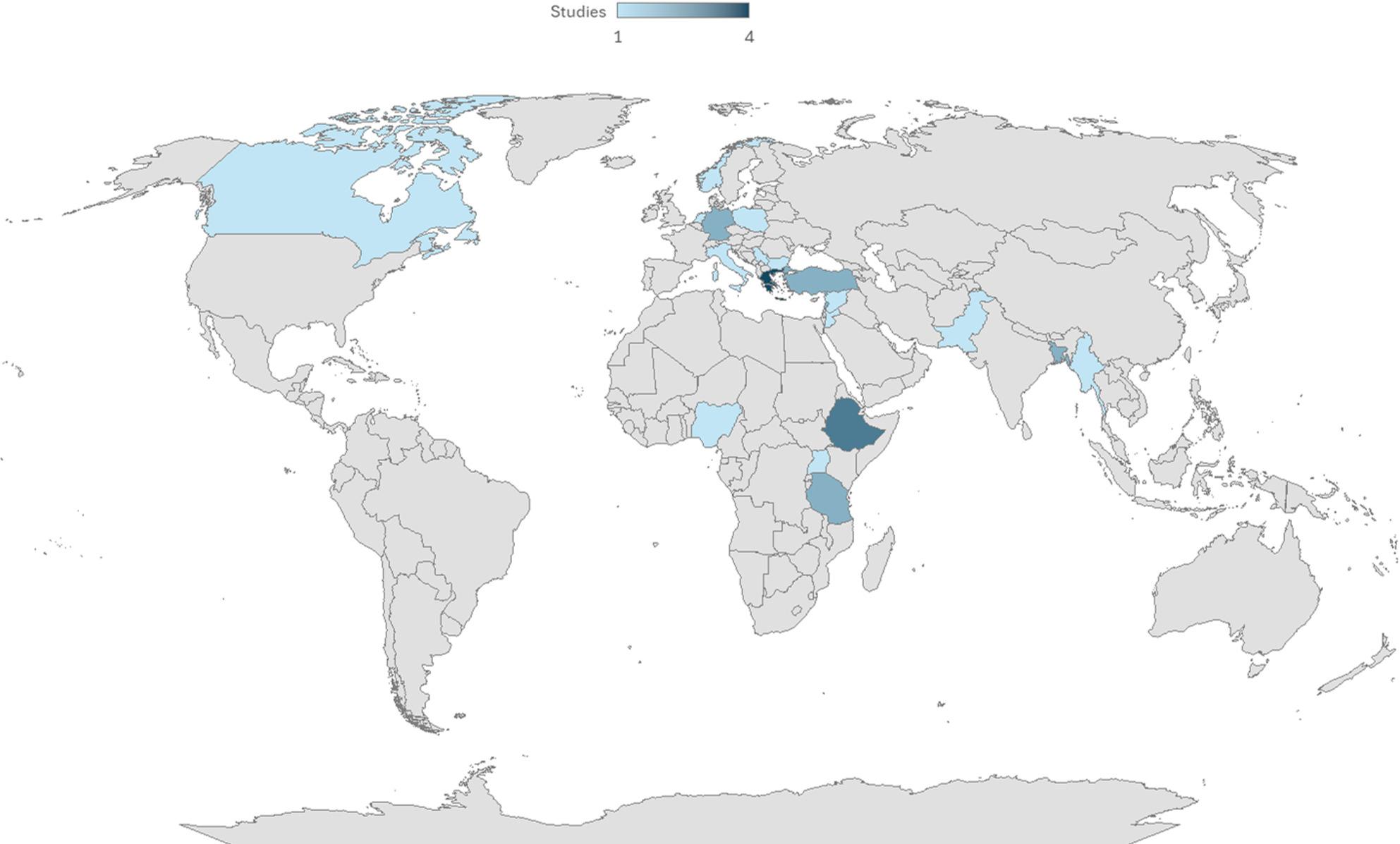
World map highlighting the countries where the selected studies were carried out

Using the World Bank’s regional classification [45], most of the interventions were implemented in Europe and Central Asia (46%), followed by Sub-Saharan Africa (25%), the Middle East and North Africa (11%), South Asia (11%), North America (4%), and East Asia and the Pacific (4%) (Fig. 2).

For interventions specifying the location where they were implemented, these took place mainly in refugee camps (*n* = 16), followed by reception centers for asylum seekers and refugees (*n* = 4), camps for internally displaced persons (*n* = 3), shelters (*n* = 4; including a university campus and orphanage set up as shelters), a transit camp (*n* = 1), and a school (*n* = 1).

### Characteristics of the participants in the interventions

All but one intervention directly involved minors in transit. The exception was aimed at parents in transit with children up to six years of age, with the ultimate objective of promoting the well-being of the children [46]. 79% of the studies included interventions aimed at adolescents (12–17 years), 68% at grade-schoolers (5–11), 18% at preschoolers (3–4 years), 14% at toddlers (1–2 years), and 7% at babies (less than 1 year). In the interventions that were designed directly for minors, caregivers of the children also participated in eight of them [34, 35, 37, 40, 47–50].

Twenty-three of the studies included interventions explicitly implemented with populations located outside their country of origin. Thirteen of the studies included interventions that benefited migrants, refugees, or asylum seekers from the Middle East (Afghanistan, Iran, Iraq, Kurdistan, Lebanon, Palestine, Syria, and Turkmenistan) [36–38, 42, 44, 46, 48, 50–55], six from East Africa (Burundi, Eritrea, and Somalia) [34, 35, 38, 39, 41, 47], two from Eastern Europe (Ukraine) [56, 57], two from Southeast Asia (Myanmar) [40, 58], one from North Africa (Algeria and Western Sahara) [38], and one from South Asia (Pakistan) [51]. Five of the interventions targeted internally displaced populations in Myanmar [49], Nigeria [59], Pakistan [60], Syria [55], and Uganda [43]. 64% of the studies explicitly mentioned the refugee status of the participants, while 11% identified the participants as asylum seekers. Three interventions specifically targeted unaccompanied minors [38, 47, 51].

For some of the interventions (32%), it was a requirement that children had specific mental health needs to be eligible, including certain diagnoses (such as post-traumatic stress disorder (PTSD) [42, 43, 55]) or symptoms (such as elevated symptoms of trauma, internalizing symptoms, or externalizing symptoms, as well as common symptoms of distress [34, 35, 39, 47, 59, 60]).

### Cultural adaptation of interventions

Although most of the interventions did not explicitly mention if the design of their different components had been carried out according to the cultural context of the target population (*n* = 17), those that did note it had considered aspects such as shared knowledge, language, behaviors, or cognitive constructs (belief, norms, etc.) in their designs (*n* = 11) [34, 35, 39, 40, 42, 44, 47, 48, 50, 52, 58].

### Approaches used by interventions

The categorization was performed using the work of Macdonald G. et al. as a reference [61]. Interventions were classified into different groups according to the basis of the approaches used, including psychoeducational, counseling, playful activities, cognitive-behavioral therapy, relationships with caregivers, pharmacological, and group work. Most interventions used multiple strategies in combination.

#### Psychoeducational

Nine studies included interventions with a psychoeducational component. The interventions employed psychoeducation to help child participants better understand and manage their emotions and thoughts, promote their autonomy, and reduce the stigma associated with mental health conditions [34, 35, 37, 40, 43, 47, 50, 51, 56]. In five of them, psychoeducation was extended to other members of the community as well, such as caregivers and teachers [34, 35, 40, 47, 50]. Two of the interventions used storytelling as a psychoeducational tool [37, 56].

#### Counseling

Some interventions employed mental health counseling (*n* = 4), where children discussed emotional and personal issues with the counselor, who then provided strategies for managing mental distress and guidance in decision-making [40, 41, 47, 59]. Counseling was individual or in groups, sometimes trauma-focused, and usually delivered by community members who were trained and supervised by mental health professionals.

#### Playful activities

Three of the interventions had an approach based on artistic activities [36, 60, 62], two on sports activities [52, 63], and two on play [54, 63]. Artistic activities included drawing, dancing, singing, and playing instruments. Sports activities included soccer, basketball, volleyball, and endurance and strength exercises. Sport and group games were used to release stress and bodily tension but also to promote autonomy, respect, and cooperation.

#### Cognitive-behavioral therapy

Many of the studies included interventions with typical components of cognitive-behavioral therapy (*n* = 16), with the aim of helping participants modify thoughts and behaviors that could cause them mental distress. The interventions focused on behavior modifications [38, 48, 60], mindfulness practices [53, 54], social skills training, including problem-solving [34, 35, 40, 44, 49, 50, 57–59], and the technique of “Eye Movement Desensitization and Reprocessing” (EMDR) [42]. Another employed the “Common Elements Treatment Approach” (CETA), which is also based on the principles of cognitive-behavioral therapy [39].

#### Relationships with caregivers

Various studies included interventions with a component of parental training that was focused on relationships with caregivers, typically defined as the mothers and fathers of the children (*n* = 6) [34, 35, 44, 46, 48, 49]. In general, these interventions were intended to positively influence parenting behaviors and indirectly benefit the well-being of children. Different types of dynamics were included, from case studies, focus group discussions between parents, or role plays. The goals were to strengthen caregivers’ ability to cope with stress, to show affection and support to their children, and to promote positive communication and nurturing interactions within the family.

#### Pharmacological

Only one of the interventions was focused on pharmacological therapy, specifically for the treatment of children with PTSD through the provision of propranolol followed by a re-enactment of the traumatic event [55].

#### Group work

Most interventions used a group approach as a mechanism to promote the psychosocial well-being of the participants (*n* = 23). Only five of the interventions utilized an individual approach, either by the use of tailored therapies (one based on the CETA intervention [39], one based on the use of EMDR [42] and a pharmacological intervention with propranolol [55]), or by the use of digital tools (a digital mental health intervention focused on problem-solving [57] and the online adaptation of a psychoeducational book [37]).

### Timing of interventions

Of the interventions with a specific duration, the range was from 1 to 21 weeks, with a mean of five weeks (*n* = 15). Considering criteria in Eapen V. et al. [64], according to which brief interventions are those of eight weeks’ duration or less, half of the interventions included in the review fall into this category. Interventions with a specific number of sessions had 1 to 21 sessions, with a mean of eight sessions (*n* = 18). Some of the interventions did not have a defined duration or number of sessions but were offered on a regular basis for those interested in attending (*n* = 8) [36, 40, 41, 44, 47, 52, 62, 63].

### Facilitation of interventions

The training level for facilitators varied, with seven interventions not requiring any type of specific training [34, 41, 44, 48, 49, 53, 63], twelve requiring some targeted non-higher education training [35, 36, 38–40, 43, 46, 47, 52, 54, 58, 59], and eight obligating facilitators to have completed higher education in topics related to the approach of the intervention, such as psychology, social work, or education [42, 50, 51, 55–57, 60, 62]. In many cases, facilitators were required to either have experience working with the target population of the intervention (or on interventions of the same nature) (*n* = 5) [34, 41, 49, 54, 63] or be from the beneficiary population itself (*n* = 7) [34, 42, 44, 47, 58, 59, 63]. Multiple interventions included training of facilitators (*n* = 9) [34, 35, 41, 43, 48, 49, 53, 62, 63], and some explicitly mentioned supervision by people who were more experienced or with a higher level of education (*n* = 8) [35, 38, 40, 41, 47, 52, 54, 62].

### Type of evaluations

43% of the studies included an evaluation of the intervention process (i.e., judgment on whether the intervention had been delivered as planned [65]), while 78% included summative evaluation of some aspect of the intervention (i.e., judgment on whether the program had achieved the initial objectives [65]). Several studies (*n* = 7) combined process and summative assessment. A summary of the characteristics of the evaluations and their results can be seen in Table 3.

**Table 3:** Characteristics of the evaluations, implementation barriers, and enabling factors for implementation

Author and reference	Title	Type of evaluation	Methodological approach	Sample size/number of beneficiaries	Evaluation results	Implementation barriers	Enabling factors for implementation
Adamkis [52]	Promoting physical activity for mental health in a refugee camp: the Skaramagas project	Process and summative evaluation: Descriptive design.	Qualitative.	269 minors.	A climate of acceptance, diversity, and gender equality was created through positive participation in physical activity. Participants reported improvements in their mental health and psychosocial well-being.	The mix of cultures and genders in the groups.	Not included.
Akoyunoglou et al. [36]	Community music therapy with refugee children in transit camps on the Greek island of Chios: 'Like one family, together'	Summative evaluation: Descriptive design.	Qualitative.	Not included.	Participants reported greater well-being and social inclusion thanks to participation in the sessions.	Lack of space. Need for flexibility due to the very fluctuating flow of volunteers, facilitators and participants (from few to 90), and the energies of the groups very different depending on the conditions of arrival on the island. Distrust of the authorities due to the discontent of the local population, which led to greater administrative restrictions.	Not included.
Alem et al. [47]	Programming to Address Suicidal Behaviour among Unaccompanied Refugee Minors in a Camp Setting: A Field Report from Ethiopia	Summative evaluation: Pre-post design without control.	Mixed methods.	Beneficiaries of psychoeducation sessions: 3436 minors. Counselling services: 1174 minors.	After the trainings and psychoeducation, it was observed that the caregivers used the material they learned and worked from a child-centered approach. Symptoms of psychological, somatic, and/or behavioral distress were reduced in 92.5% of the children receiving counseling at six months.	The change of the team in the collaborating organizations. Combat existing stigma. As caregivers had their own experiences of trauma and chronic stress, effort had to be put in so that, in addition to providing them with tools to help the children, they could manage self-care and their emotions.	The participation of caregivers. The use of narratives in psychoeducation/counseling sessions facilitated the connection with the participants. The presentation of information in the form of dramatized sketches on the radio facilitated the retention of messages. The incorporation of aspects of the local belief system into the counseling was helpful, as was the normalization and inclusion of culture-specific experiences. The use of creative methods to express complex concepts to children and not exclusively verbal according to the stage of development facilitated the sessions. Gender groups helped participants feel freer and more confident to share their experiences.
Bleile et al. [63]	Process evaluation of TeamUp: a movement-based psychosocial intervention for refugee children in the Netherlands	Process evaluation.	Mixed methods.	2183 minor beneficiaries. In the evaluation: 24 facilitators, 94 minors, and 10 staff members.	Attendance and group sizes were lower than expected. The fidelity of the facilitation of the intervention was moderate to adequate (showing a need for strengthening), while the competencies of the facilitators were high. Facilitators expressed high levels of satisfaction, self-efficacy and support, as well as a low workload. The intervention was perceived positively by the different actors, with a positive impact on the psychosocial learning and well-being of the participants.	Difficulties regulating children's energy and challenging behaviors.	Not included.
Borja Jr. et al. [40]	Child-centred, cross-sectoral mental health and psychosocial support interventions in the Rohingya response: a field report by Save the Children	Process and summative evaluation: Descriptive design.	Mixed methods.	Not included.	Caregivers and children reported feeling safe in child-friendly spaces. They articulated that the physical space helped them improve their mental health and psychosocial well-being. In addition, quantitative data showed that 8 out of 10 adolescents aged 11-17 years and 7 out of 10 children aged 4-10 years had improved their well-being compared to the previous year.	The lack of a mental health expert and consistent psychosocial support made it difficult to develop the comprehensive response on the ground. It was difficult to find qualified personnel to provide such services in the region, in part due to the need to speak Rohingya or Chittagonian. Ongoing personnel changes. Physical barriers to accessing services. A changing humanitarian crisis.	Not included.
Callaghan et al. [58]	Fostering Prosociality in Refugee Children: An Intervention With Rohingya Children	Summative evaluation: Pre-post design without control.	Quantitative.	152 minors.	The intervention protocol resulted in significant positive changes in several of the prosocial behavioral variables and related cognitive-affective processes.	Not included.	Not included.
Clement-Lawrence et al. [59]	Trauma-focused counselling and social effectiveness skills training interventions on impaired psychological functioning of internally displaced adolescents in Nigeria	Summative evaluation: Pre-post design with control.	Quantitative.	60 minors.	The two interventions evaluated significantly improved the psychological functioning of adolescents.	Not included.	Not included.
de Freitas-Girardi et al. [62]	Creative expression workshops as Psychological First Aid (PFA) for asylum-seeking children: An exploratory study in temporary shelters in Montreal	Process evaluation.	Qualitative.	Not included.	It helped promote emotional security and a sense of normalcy in children and supported the creation of connections between children and parents. The expression of past and present experiences in the workshops was seen as a way to promote self-efficacy in children and was reported as a way to give comfort and hope in a time of high instability.	Many unmet needs in minors due to lack of resources in shelters. Fluctuating number of young people, experiencing difficulty in containment when they were large groups. A feeling of helplessness on the part of facilitators who were unable to provide individualized attention to children who needed it.	The implementation of monthly supervision for facilitators helped them overcome their emotions of helplessness and frustration.
El-Khani et al. [48]	Assessing the Feasibility of Providing a Family Skills Intervention, “Strong Families”, for Refugee Families Residing in Reception Centers in Serbia	Process and summative evaluation: Pre-post design without control.	Mixed methods.	25 families, 25 minors.	Reduction in total difficulties after participation in the program. Specifically, emotional problems decreased, as well as behavioral problems and hyperactivity. Improvements in parenting practices and family adjustment skills were also observed. According to qualitative data, parents experienced improvements in parenting self-efficacy, reduced physical punishment, prioritized childcare, improved communication with their children and partners, considered intervention as culturally acceptable, helped them identify the needs of others, and generally found it helpful.	Lack of space and interpreters (Serbian to Dari) prevented both parents from participating in the workshop.	Good connection to other services allowed referral for external support when required by caregivers or their children.
El-Khani et al. [49]	Implementation of a Family Skills Programme in Internally Displaced People Camps in Kachin State, Myanmar	Process and summative evaluation: Pre-post design without control.	Quantitative.	100 families. Parents with a maximum of two children aged 8-15 years.	Improvements in children's mental health, parenting practices, and adjustment skills in parents and families.	Little participation from parents, who worked outside.	Not included.
Ertl et al. [43]	Community-Implemented Trauma Therapy for Former Child Soldiers in Northern Uganda A Randomized Controlled Trial	Summative evaluation: Randomized controlled trial.	Quantitative.	85 minors.	Greater reduction in severity of PTSD symptoms in the group with narrative exposure therapy. Also, improvement in other secondary aspects, such as depression, suicidal ideation, feelings of guilt, stigmatization, and functioning.	Not included.	Not included.
Fine et al. [34]	Improving mental health in low-resource settings: A feasibility randomized controlled trial of a transdiagnostic psychological intervention among Burundian refugee adolescents and their caregivers	Process and summative evaluation: Cluster randomized controlled trial.	Mixed methods.	82 minors and 64 caregivers.	Significant decrease in psychological distress in both groups. Significant decreases in internalizing symptoms in both groups and somatic discomfort in the intervention group. Parental involvement increased in the intervention group, as well as decreased corporal punishment. Positive parenthood was reduced in the control group. Regarding the evaluation of the process, the sessions were implemented with high fidelity, as well as high levels of adherence. According to qualitative data, the intervention showed good feasibility, relevance, acceptability, and safety. Most minors reported using the strategies learned in the intervention to deal with problems at home. Caregivers reported changes in their children, such as greater initiative at home, more discipline, well-being, and the use of relaxation exercises. They also reported changes in themselves, such as better relationships with their children, greater ability to counsel their children, and use of discipline techniques other than physical punishment.	Conflicts with school schedules to attend sessions. Difficulties in recruiting facilitators and retaining them.	Not included.
Foka et al. [53]	Promoting well-being in refugee children: An exploratory controlled trial of a positive psychology intervention delivered in Greek refugee camps	Summative evaluation: Pre-post design with control.	Mixed methods.	72 minors.	Improvements in well-being, self-esteem, optimism, and depression symptoms in the intervention vs. control group. Focus group participants highlighted the importance of the intervention in developing a sense of community and building its strengths.	Difficulty in accessing refugee camps to facilitate intervention and conduct research. Access to children limited to a few hours a day outside of the summer months. There was no capacity to include all interested people in the program. Many of the children took their parents to the sessions spontaneously and the program was not prepared to serve them.	Not included.
Gotseva-Balgaranova et al. [50]	Impact evaluation of the evidence-based trauma stabilisation programme for refugee, asylum seeking and immigrant families	Summative evaluation: Pre-post design without control.	Quantitative.	15 minors and 16 parents.	The children experienced a reduction in all PTSD symptoms after participating in the program, as measured by self-report and parent reporting. There was a significant reduction in reported intrusive episodes, while anxiety levels remained almost unchanged (according to self-reports). The children's levels of depression, agitation, and dissociation decreased significantly (parents' report). Parents experienced a moderate reduction in their depression symptoms, although not significant.	Not included.	Not included.
Gul et al. [60]	Management Of Self-Concept, Disruptive Behavior And Aggression Through Art And Behavior Therapy Among Internally Displaced Children	Summative evaluation: Pre-post design with control.	Quantitative.	60 minors.	Improvement of self-concept and significant decrease in anger and disruptive behavior in the children who received the intervention greater than those in the control group.	Not included.	Not included.
Kalantzi-Azizi et al. [37]	Presentation of an online programme for children refugees: Pilot study in Greece	Process evaluation.	Qualitative.	6 minors.	Parental engagement improved, children responded positively, and psychological difficulties were identified earlier. The authors concluded the feasibility of using this manual on Afghan refugees in Greece.	Not included.	Not included.
Kuru et al. [54]	Social-emotional outcomes in refugee children: A pilot randomized controlled trial of a school-based mindfulness intervention implemented in a refugee camp	Summative evaluation: Single-blind randomized controlled trial.	Quantitative.	76 minors.	Improvement of social skills, self-esteem, and resilience over time for the group with the intervention. Reductions in social and emotional problems in children with high levels of introversion in the pre-test.	Problems negotiating the implementation of the intervention in the refugee camp, requiring strong connections with public officials and administrators of the camps. A school-wide approach could not be used due to security issues, schedules, and operational dynamics in the field.	Same language of facilitators and participants. The fact that it was implemented in schools made it easier for children to meet at specific times with the help of teachers.
Lakkis et al. [46]	A Pilot Intervention to Promote Positive Parenting in Refugees from Syria in Lebanon and Jordan	Summative evaluation: Pre-post design without control.	Quantitative.	Caregivers of 65 minors.	Perceived difficulties, behavioral problems, and hyperactivity in children aged 3-6 years were significantly reduced, as well as dysfunctional interactions of parents with their children. Parental mental health and well-being improved, as well as improvements in parenting were reported, reducing practices such as ignoring, shaming, or physical punishment.	Not included.	Not included.
Melogno et al. [56]	War refugee children: an intervention based on coping strategies	Process and summative evaluation: Descriptive design.	Qualitative.	15 minors.	The children showed very active participation and appropriate understanding of the experience. Each session they had greater independence. They respected the instructions at all times. The intervention fulfilled the long-term objective of promoting resilience attitudes. The children spontaneously resorted to the "three verbs" strategy.	Not included.	Not included.
Metzler et al. [41]	Educational, psychosocial, and protection outcomes of child- and youth-focused programming with Somali refugees in Dollo Ado, Ethiopia	Summative evaluation: Cluster-randomized controlled trial.	Mixed methods.	1571 minor beneficiaries. 195 participants in evaluation.	Reduction in difficulties, increase in prosocial behavior, and gains in assets for development (internal and external coping resources available to the child). This difference was especially marked in children aged 6-11 years in the intervention group (greater decrease in difficulties) vs. in the control group.	Full capacity in centers.	Not included.
Meyer-Demott et al. [38]	A controlled early group intervention study for unaccompanied minors: Can Expressive Arts alleviate symptoms of trauma and enhance life satisfaction?	Summative evaluation: Pre-post design with control.	Quantitative.	145 minors.	Increased life satisfaction and hope for the future in the intervention group.	Dropout of participants in the follow-up period.	Not included.
Murray et al. [39]	An evaluation of a common elements treatment approach for youth in Somali refugee camps	Process and summative evaluation: Pre-post design without control.	Mixed methods.	37 caregiver-child or adolescent dyads.	Children and caregivers reported a decrease in symptoms of internalization, externalization, and post-traumatic stress, as well as improvements in well-being. The qualitative results were positive towards the acceptability and relevance of the treatment, as well as its feasibility.	Some caregivers did not commit to attending the sessions or that of their children. The participants made requests for material things to the implementers during the sessions. Religious barrier, since part of the population preferred religious-based solutions for people with psycho-emotional difficulties.	Facilitators reported that post-training practice groups and supervision helped them better understand the intervention material. Having quality materials (manuals) helped them facilitate the intervention.
Nakkash et al. [44]	Process evaluation of a community-based mental health promotion intervention for refugee children	Process evaluation.	Mixed methods.	150 minors.	Overall, the objectives of the sessions were achieved, and the specific activities of each session were implemented as planned. The children indicated high satisfaction with the sessions.	Difficulty in attending to mentors, mostly young university students. The high number of facilitators made it difficult to replicate, each with their own personal style. The escalation of a conflict in a neighboring country made it difficult for the participants to concentrate. Difficulty in having a local youth coalition that was collaborating in the observation of the sessions and had to be present during their entirety.	There was a very detailed implementation manual. The implementation team attended intensive training. High commitment of the implementation, research, and field teams.
Perilli et al. [42]	EMDR group treatment of children refugees-A field study	Summative evaluation: Pre-post design without control.	Quantitative.	8 minors.	Decrease in severity of post-traumatic symptoms.	Difficulty in finding translators. High mobility of refugees, who abandoned the intervention.	Not included.
Scheiber et al. [51]	Resilience training for unaccompanied refugee minors: A randomized controlled pilot study	Summative evaluation: Randomized controlled trial.	Mixed methods.	55 minors.	No change in symptoms of trauma-related disorders. Intervention group reported an increase in well-being after completing the program. Qualitative data indicated positive changes in functionality.	Not included.	Not included.
Singh et al. [35]	Cultural adaptation of a scalable psychological intervention for Burundian refugee adolescents in Tanzania: a qualitative study	Process evaluation.	Qualitative.	80, minors and parents.	Acceptable, understandable, and relevant intervention for participating adolescents and caregivers.	Participation of non-professional refugee workers in facilitation, facing administrative barriers to paid work.	Design according to priorities identified by the beneficiary population; adapted to low levels of literacy, language, and context. Inclusion of a protocol for the management of cases of sexual violence. The effort made in simplifying processes for suppliers and beneficiaries.
Thierrée et al. [55]	Trauma reactivation under propranolol among traumatized Syrian refugee children: preliminary evidence regarding efficacy	Summative evaluation: Open-label clinical trial.	Quantitative.	117 minors.	A clinically relevant reduction in PTSD symptoms and depression was observed in all post-treatment measures compared to baseline levels (at 4 and 13 weeks post-treatment). This was not the case for anxiety symptoms, which were reduced to return to their original state.	Loss of follow-up of participants probably due to bombings near the refugee camp.	The high social acceptability facilitated implementation.
Weisz et al. [57]	Effects of a brief digital problem-solving intervention on depression and anxiety symptoms in Ukrainian children and adolescents displaced by war: a crossover, randomised controlled trial	Summative evaluation: Crossover, randomized controlled trial.	Quantitative.	709 children and adolescents.	Reduction of internalizing symptoms.	Post-intervention assessment was not possible for multiple students moving to new refugee spaces or returning to their country of origin. Little collaboration from caregivers, who had to offer their perspective on improvements in their children.	Not included.

EMDR: Eye Movement Desensitization and Reprocessing; PFA: Psychological First Aid; PTSD: Posttraumatic Stress Disorder

Among the evaluations with a summative component (*n* = 23), four employed a descriptive design, without indicating the quantitative differences between the pre- and the post-intervention mental health and psychosocial well-being [36, 40, 52, 56]; nine assessed the quantitative differences between the pre- and the post-intervention mental health and psychosocial well-being without a control group [39, 42, 46–50, 58], including one open-label clinical trial [55]; four assessed the pre-post quantitative differences with a control group but without randomization of participants between groups [38, 53, 59, 60]; and six evaluated the pre-post quantitative differences with a control group and randomization of participants, following a randomized controlled trial design [34, 41, 43, 51, 54, 57].

### Methodological approaches

Six of the studies used a qualitative methodology [35–37, 52, 56, 62], twelve quantitative [38, 42, 43, 46, 49, 50, 54, 55, 57–60], and ten mixed-methods [34, 39–41, 44, 47, 48, 51, 53, 63].

### Sample size/number of beneficiaries

All included studies (except three [36, 40, 62]) mentioned the sample size used in the evaluation or the number of beneficiaries of the interventions. In those that included the number of beneficiaries, these ranged from a small group of six minors [37] to a total of 3436 minors [47], with an average of 80 participants.

### Results of the evaluations

In relation to the implementation process, several studies identified positive aspects (*n* = 10), such as high fidelity [34, 44], high levels of adherence [34], good feasibility [34, 37, 39], relevance [34, 35, 39], acceptability [34, 35, 39, 48, 56], safety [34, 40], as well as high levels of satisfaction of both the beneficiaries [44, 62, 63] and the facilitators [63]. One intervention reported low adherence and moderate fidelity [63].

Regarding the evaluation of the effects of interventions on children, all studies reported some degree of positive change, although with varying degrees of strength, considering different sample sizes and study designs (with a high proportion of studies without control groups or without random assignment of participants). Changes included an overall improvement in mental health and psychosocial well-being [34, 36, 39, 40, 47, 49, 51–53, 63], the overall reduction of internalizing [34, 39, 57] and externalizing [39, 47] symptoms, the reduction of somatic discomfort [34, 47], reduction of difficulties, including emotional, behavioral, and hyperactivity problems [41, 46, 48], decreased anger and disruptive behavior [60], increased resilience [54, 56], increased optimism [38, 53, 62], increased initiative, discipline, and autonomy [34, 56], gain of internal and external coping resources [41, 62], improved psychological functioning [43, 51, 59], increased life satisfaction [38], increased self-esteem [53, 54, 60], reduced symptoms of depression [43, 50, 53, 55], reduced symptoms of post-traumatic stress [39, 42, 43, 50, 55], reduced thoughts of suicidal ideation [43], increased prosocial behavior [36, 41, 52, 54, 58], improved social skills [54], and a greater sense of community [53].

Regarding the impact on the parents or other participating caregivers, various improvements were also reported, both in their parenting practices and in their well-being (*n* = 8). In parenting, some studies reported an increase in parental involvement [34, 37], improved parental self-efficacy [48], prioritization of childcare [47, 48], improved communication between parents and children [48], greater ease in identifying the needs of their children [37, 48], improvement in parenting practices and family adjustment skills in general [48, 49], decrease in corporal punishment [34, 46, 48], decrease in practices such as ignoring and shaming [46], and improvement in parents’ relationships with their children [34, 46, 62]. Two studies also reported an improvement in parents’ mental health and psychosocial well-being because of their participation in the intervention [46, 50].

Three studies reported sustained use of the tools provided during the intervention post-completion in both children and their caregivers [34, 47, 56].

### Implementation barriers

Most studies mentioned implementation barriers (*n* = 19) (Table 3), including the distrust of the authorities of the implementation site/administrative restrictions [35, 36, 53, 54]; physical barriers experienced by beneficiaries to attend interventions [40]; lack of physical space to carry out interventions [36, 48]; unpredictability of humanitarian crises that affects population flows [36, 40, 62] and the ease of concentration of participants [44]; low availability of facilitators and their rotation [34, 36, 40, 42, 44, 48]; changes in partner organizations [47]; lack of capacity to include all stakeholders in interventions [41, 53, 54]; unwillingness of parents to participate in interventions for work reasons [49, 57] or minors for school activities [34, 53, 54]; difficulties of having participants with heterogeneous sociodemographic and cultural characteristics [39, 52]; stigma surrounding mental health diagnoses and treatment [47]; mental health and psychosocial well-being difficulties of the parents themselves [47]; challenging behaviors of the minors [63]; frustration of the implementing team for not being able to fully meet the material and psychosocial needs of the participants [39, 62]; as well as the abandonment of the participants, often due to the mobility situation in which they found themselves [38, 39, 42, 55, 57].

### Enabling factors for implementation

Only eight studies mentioned aspects that would have acted as facilitators of the implementation of the intervention (Table 3), including the cultural adaptation of the intervention during the design phase, incorporating aspects of the local belief system and language, as well as the inclusion of culture-specific experiences [35, 47, 54, 55]; the adaptation of interventions according to the stage of development of the beneficiaries [47]; the implementation of separate sessions for each gender [47]; the use of effective means of communication in the context of implementation, such as radio [47]; the implementation of interventions in the school space, taking advantage of a space and time where children already meet naturally and can count on the support of teachers [54]; good connection with other services to refer participants with needs that the intervention could not address [35, 48]; having clear reference manuals for facilitators, as well as having exhaustive training and regular sessions for the consolidation of the knowledge received in these [39, 44]; the development of supervision sessions with the facilitators on a regular basis as a psycho-emotional containment strategy [62]; and the motivation and commitment of the field team [44].

## Discussion

This scoping review effectively identifies evidence from the last fifteen years on interventions that promote the mental health and psychosocial well-being of migrant, refugee, and asylum-seeking children and adolescents in transit globally, the vast majority published in the last five years. Most of the interventions had been implemented in refugee camps and were aimed at forcibly displaced adolescents and school-age children. In general, these interventions sought to promote the mental health and psychosocial well-being of the minors without addressing specific health conditions, combining different approaches, such as elements of cognitive behavioral therapy and psychoeducation, fostering good relationships between minors and their parents, and using a group format. All included studies reported some degree of positive change; however, the strength of evidence varied considerably across study designs and sample sizes.

### Nature of the interventions

Our scoping review differs from other literature reviews that focus exclusively on minor refugee populations of specific ages and/or living in high-income countries, on effectiveness studies published only in peer-reviewed journals or, above all, without specifically considering the characteristics of the transit context [66–74]. One of the main challenges in the transit context is the limited contact between teams implementing mental health and psychosocial well-being interventions and the population of minors on the move, generally limited to a series of sporadic and transient contacts in refugee camps, reception centers for asylum seekers, or transit centers [75]. This makes it difficult to adhere to interventions, as indicated by some studies in our review [38, 39, 42, 55, 57]. Another major challenge posed by the transit context is exposure to stressors typical of this phase of the migratory journey, such as unstable housing, severe material deprivation, threats to safety, inconsistent access to education, protection and health care services, family separation or the risk of it, limited access to reliable information, barriers related to language and documentation, and the emotional burden of repeated displacement can negatively affect the mental health and psychosocial well-being of children and adolescents [76–78]. Taking this into consideration, our review identifies the following interventions as particularly relevant to the transit context: brief, group-based, trauma-informed [36–39, 43, 47, 50, 51, 55, 59, 60], and strength-based interventions [48, 51, 53, 62, 63], as well as those that include psychoeducation, social skills and problem-solving training, and parenting training for caregivers. Previous literature reviews, focusing mainly on refugee and asylum-seeking children and adolescents in camps and settlements, have also concluded that it is important to use family-based approaches when promoting the psychosocial well-being of this population [70, 73], the benefits of using a group facilitation format, promoting the creation of bonds between the participants [66, 67, 70], and the usefulness of trauma-informed cognitive behavioral therapy-based tools in promoting the mental health of these populations [66, 67, 70, 71, 74]. Overall, these findings reinforce the core Inter-Agency Standing Committee (IASC) guidance that, in contexts of mobility and transit, interventions should avoid overly clinical or diagnostic approaches and instead prioritize scalable, resilience-focused psychosocial support [79]. This underscores that what is most effective in transit settings are flexible, non-specialized interventions that restore safety, predictability, connection, and agency. Rather than addressing individual pathology, these approaches leverage existing strengths and protective factors, aligning with the IASC emphasis on community-based, non-clinical mental health and psychosocial well-being support that can be rapidly deployed and sustained in highly fluid environments [79].

### Origin of participants and implementation locations

Most of the interventions identified in our review served populations from the Middle East displaced by the wars and political instability that have affected the region in recent decades. Two of the countries most affected by these conflicts, whose populations are particularly well represented in our study, are Syria, which has been in civil war since 2011, with an estimated five million refugees in other countries and six million internally displaced persons [80], and Afghanistan, where more than 40 years of conflict, natural disasters, poverty, food insecurity, and the Taliban takeover in 2021 have led to nearly six million refugees in different countries [81]. Our study also highlights the populations of Somalia, Myanmar, and Ukraine among the main beneficiaries of interventions. In Somalia, clashes between the government and various insurgent groups, coupled with climate-related disasters and famine, have led to the mass displacement of its population for years, with over five million refugees and asylum seekers worldwide by the end of 2024 [82]. In Myanmar, the outbreak of violence against the Rohingya Muslim ethnic minority in 2017 led to the exodus of this population from the country, with nearly one million stateless refugees [83]. In Ukraine, following the start of the Russian invasion in 2022, a large part of the population has been displaced to other areas, with nearly four million internally displaced persons and nearly seven million refugees at the beginning of 2025 [84].

Multiple countries where the interventions included in our review were implemented are located on the main migration routes of populations displaced by war in the Middle East, including Turkey, Lebanon, and Jordan in the Middle East, and Greece, Bulgaria, and Germany in Europe [85]. Ethiopia, the setting for three of the studies included, is one of the main countries of refuge for people displaced by violence and climate disasters in the Greater Horn of Africa [86], while Bangladesh, where two of the studies were conducted, is the main destination for Rohingya displaced persons from Myanmar [83].

It is important to note that our study did not identify two of the populations with the highest levels of displacement in the study inclusion period, those of Venezuela and South Sudan [87]. Furthermore, no interventions were identified in the Latin American and Caribbean region, which has experienced a considerable increase in the international migrant population in the first two decades of the 21st century, rising from 24.6 million in 2000 to 42.9 million in 2020 [88], due to causes such as economic and political conflicts, violence perpetrated by organized crime or guerrilla groups, and natural disasters in the region [89, 90].

### Lack of specific interventions for vulnerable subgroups

As mentioned above, most of the children and adolescents served by the identified interventions had been forcibly displaced due to various humanitarian crises, particularly armed conflicts, with harmful short- and long-term effects on their health, well-being, education, and socio-emotional development [91]. In addition, most of these minors have been exposed to different stressors throughout their displacement which can have detrimental effects on their mental health and psychosocial well-being [92–95]. However, the experiences of forcibly displaced minors, as well as how these experiences are processed and their impact, will depend on factors such as ethnic, religious, gender and sexual identity, the presence of any disability, the minor’s stage of development, their previous experiences and coping skills, their socioeconomic and family context, and the broader protection environment, including access to services and exposure to discrimination or insecurity [91]. Considering this, one of the main limitations we identified in our literature review is the lack of specific interventions for subgroups in situations of greater vulnerability, such as minors with disabilities, those belonging to discriminated ethnic and religious minorities, girls, or members of the LGBTQ+ community, who face additional challenges that are overlooked when considering only the general population. Only two of the interventions focused on a particular ethnic-religious group [40, 58], two had a gender perspective [47, 51], three considered the specific situation of unaccompanied minors [38, 47, 51], and eleven had broadly included aspects of the participants’ cultures in the interventions [34, 35, 39, 40, 42, 44, 47, 48, 50, 52, 58].

### Limited report on the cultural adaptation process

It is important to note that, although many interventions did not explicitly include a cultural adaptation process, we cannot rule out the possibility that some of them did carry out such a process even though they did not mention it in the study, given the widespread underreporting in the literature of the cultural adaptation process in evaluations of interventions promoting mental health and psychosocial well-being [96]. Precisely to remedy the lack of evidence on this process, efforts have recently been made, such as the development of a guide to systematize the reporting of the cultural adaptation of psychological interventions used in clinical trials, the RECAPT (Reporting Cultural Adaptation in Psychological Trials) [96]. In interventions such as those included in most of the studies in this review, where psychotherapeutic approaches developed in Western contexts are used, it is essential to carry out cultural adaptation, as these are based on theories that may conflict with the beliefs of participants with a non-Western background [97]. One of the psychotherapies where adaptation becomes particularly relevant is cognitive-behavioral therapy, as it involves identifying and modifying beliefs, underlying assumptions, and automatic thoughts that cause psychological distress, which will vary depending on the culture [98, 99]. However, half of the studies identified in this scoping review that use this approach do not explicitly report a cultural adaptation process. Although there is diverse evidence on the role of cultural adaptation in improving the acceptability and effectiveness of interventions aimed at promoting mental health and psychosocial well-being [100–103], improving the documentation of cultural adaptation processes in interventions aimed at migrant and refugee minors in transit will contribute to understanding the effects of this process on the effectiveness of treatment, feasibility, and acceptability of interventions targeting this population [96].

### High variability between studies limits their comparability

Another important aspect to highlight from the studies identified is the wide variability in their design, which limits their comparability. Differences include the type of evaluation, methodological approach, and sample size, resulting in varying strengths of the conclusions reached. Differences were also observed in the multiple outcomes evaluated, including aspects as varied as prosocial behavior, psychosocial functioning, changes in parenting practices and parental adjustment, resilience, symptoms of PTSD, depression and anxiety, well-being, optimism, and self-esteem, which makes it difficult to compare the effectiveness of the different interventions implemented. Even for studies analyzing the same outcome, different studies used different tools to measure it (generally presenting good psychometric properties for the study population).

### Factors for improving the implementation of interventions

With a view to future interventions promoting the mental health and psychosocial well-being of children and adolescents in transit, our study identifies a series of factors that can improve the implementability of interventions. First, as discussed above, some studies highlight the importance of tailoring interventions to the needs of different sociodemographic groups to improve their relevance and acceptability [34, 35, 38–40, 42, 44, 47, 48, 50–52, 54, 58]. Several studies also reflect the importance of adequate training and supervision of facilitators, as well as making comprehensive implementation manuals available to them, which improves implementation fidelity [39, 44, 62]. Most studies showed the feasibility of facilitation by people without higher education, in several cases from the beneficiary community itself. This is particularly relevant considering the common resource constraints in implementation contexts. Community participation also facilitates the adaptation of the program to the beneficiary population, improving acceptability and fostering a sense of ownership in the recovery and response process, rather than positioning the target population solely as recipients of humanitarian aid [104]. Some studies reported difficulties in meeting the needs of beneficiaries and their families when these went beyond the support provided by the interventions [39, 62], which other studies had managed to overcome by connecting with other services and working collaboratively with them [35, 48]. Also, the importance of having good connections with authorities and staff at implementation centers to gain their trust and limit administrative barriers to implementation [35, 36, 53, 54]. Finally, the lack of availability and space to carry out interventions, as reported by several studies [34, 36, 40, 48, 53, 54], can be limited by using times and places where children and adolescents naturally gather, such as spaces where teaching activities take place [54], improving the feasibility and adherence of interventions.

### Equity implications

The gaps identified in the review, with the absence of studies for certain geographies and vulnerable groups, as well as the omission of cultural adaptation in some interventions, reflect significant inequities in the implementation of interventions and their evaluation among different social groups. The development of evaluation projects is conditioned by the availability of resources for their execution, both financial and expertise, which are provided by donors and professionals in the field. This can lead to certain social groups and intervention approaches being prioritized according to the political and commercial interests of donors [105, 106] and the academic interests of researchers [107]. Among the projects that report their source of funding, most come from bilateral agencies and private foundations based in high-income countries. Although most of the selected projects are authored by individuals from institutions based in the study location, in multiple studies conducted in low- and middle-income countries, the first author and/or senior author of the research belongs to an institution in a high-income country. Therefore, it is important to consider the role that actors with the power to influence the prioritization of certain populations and approaches may have played in the studies identified—with a high presence of institutions from the Global North—which may be subject to their interests [108], not necessarily aligned with the mental health and psychosocial well-being needs of the migrant and refugee children and adolescents in transit.

### Limitations

Despite the important findings, our review has some limitations. First, a potential limitation is that the searches were limited to the period from 2010 to the present, which may have excluded studies relevant to the topic of study published before 2010. However, this criterion was used to identify the most relevant literature possible for the present. Second, the databases used, although sufficient according to the recommendations in the literature [25], may have led to the exclusion of studies that met the inclusion criteria but were not registered in these databases, mainly those written in languages other than English. Third, the absence of an assessment of the methodological quality of the studies included may limit the robustness of the findings. However, considering the objective of the review and in accordance with the PRISMA-ScR guidelines, this assessment is optional in scoping reviews [22].

## Conclusion

Our scoping review effectively describes the evidence available in the literature on interventions promoting mental health and psychosocial well-being among migrant, refugee, and asylum-seeking children and adolescents in transit, highlighting the variability in the design of the evaluations. This study has identified several gaps in the literature, including studies on interventions targeting some of the populations with the highest displacement during the period covered by the review, such as Venezuela and South Sudan, as well as interventions carried out in the Latin American and Caribbean region. The study also highlights the importance of future interventions and evaluations considering the particularities of different sociodemographic groups, especially those in situations of greater vulnerability, both in implementation and in the evaluation of effectiveness, as well as the importance of improving reporting on the cultural adaptation processes of interventions. Finally, our review identifies several factors that strengthen the implementability of interventions, which should be considered for future interventions to improve aspects such as fidelity, feasibility, relevance, acceptability, and adherence to eventually improve their impact on beneficiary populations.

## Supplementary Information

Below is the link to the electronic supplementary material.

Supplementary Material 1

## Data Availability

The documents supporting the conclusions of this review are available in the references section of the article.

## Abbreviations

APA: American Psychological Association
CETA: Common Elements Treatment Approach
EMDR: Eye Movement Desensitization and Reprocessing
IASC: Inter–Agency Standing Committee
JBI: Joanna Briggs Institute
LGBTQ+: Lesbian, Gay, Bisexual, Transgender, Queer, Plus other sexual orientations and gender identities
PRISMA: ScR–Preferred Reporting Items for Systematic Reviews and Meta–Analyses extension for Scoping Reviews
PTSD: Post–traumatic Stress Disorder
RECAPT: Reporting Cultural Adaptation in Psychological Trials
